# Ultrasonic-Assisted Synthesis of CdS/Microcrystalline Cellulose Nanocomposites With Enhanced Visible-Light-Driven Photocatalytic Degradation of MB and the Corresponding Mechanism Study

**DOI:** 10.3389/fchem.2022.892680

**Published:** 2022-04-06

**Authors:** Chaosheng Zhu, Xiangli Zhang, Yongcai Zhang, Yunlin Li, Ping Wang, Yanchi Jia, Jin Liu

**Affiliations:** ^1^ Zhoukou Key Laboratory of Environmental Pollution Prevention and Remediation, School of Chemistry and Chemical Engineering, Zhoukou Normal University, Zhoukou, China; ^2^ College of Chinese Language and Literature, Zhoukou Normal University, Zhoukou, China; ^3^ School of Chemistry and Chemical Engineering, Yangzhou University, Yangzhou, China; ^4^ Henan Key Laboratory of Rare Earth Functional Materials, International Joint Research Laboratory for Biomedical Nanomaterials of Henan, Zhoukou Normal University, Zhoukou, China

**Keywords:** cds, microcrystalline cellulose, visible-light photocatalysis, MB decolorization, charge separation

## Abstract

A simple and efficient ultrasonic-assisted approach was designed to synthesize CdS/microcrystalline cellulose (MCC) nanocomposite photocatalyst. The obtained products have been characterized by XRD, FE-SEM, TEM, UV-Vis DRS, and nitrogen adsorption isotherms. The results showed that the intimate contact of MCC and CdS is beneficial for enhancing the photocatalytic performance because heterojunction formation can efficiently promote the separation of photogenerated electrons and holes of the nanocomposite photocatalyst. By using 10% MCC coupled CdS, the decoloration rate of methylene blue (MB) in the solution under visible-light was increased nearly 50%. In addition, the reuse experiments confirmed that the CdS/MCC nanocomposite photocatalyst had outstanding cycle performance and durability. Mechanism study demonstrated that hydroxyl radicals, photogenerated holes and superoxide radicals were the active species in the photocatalytic oxidization degradation of MB.

## Introduction

Since TiO_2_ as a photocatalytic material for water decomposition in 1972, photocatalysis has attracted great interest and is recognized as a promising strategy for treating pollutants because it is highly effective, low cost and environmentally friendly (Liu et al., 2021; Mohua Li et al., 2020b; Ren et al., 2022; Fang Li et al., 2020). In view of the drawbacks of TiO_2_ (especially it can only utilize ultraviolet-light from the Sun) (Sheng et al., 2020; Ni et al., 2021; Jiang et al., 2020), narrow bandgap visible-light-active semiconductors such as BiOBr, BiOI, SnS_2_, CdS, g-C_3_N_4_, etc. have been increasingly studied as photocatalysts and their photocatalytic mechanisms have been explored in numerous published works (Yang et al., 2018; Wei et al., 2017; Silva-Gaspar et al., 2022; Liu et al., 2020; Beyhaqi et al., 2021). For developing efficient visible-light-active photocatalyst, CdS is one of the extensively studied semiconductor materials because its bandgap matches well with the spectrum of visible light (Cheng et al., 2018; Liu et al., 2021). However, the photocatalytic efficiency of CdS is fairly poor by reason of the fast recombination of photo-generated holes and electrons (Gurugubelli et al., 2022). Additionally, CdS nanoparticles are prone to encounter photocorrosion, namely, the surface sulfide ions can be oxidized to sulfur by photogenerated holes under the irradiation (Ning and Lu, 2020). In consideration of that photocatalytic reactions occur on the surface of CdS, necessary modifications are needed to stabilize the surface sulfide ions and to transfer the photogenerated holes out from the surface in order to suppress photocorrosion (Jiang et al., 2021). Many measures have been devoted to improving the photocatalytic efficiency and inhibiting the photocorrosion of CdS, like changing the surface structure of CdS nanoparticles by controlling morphology (Liu et al., 2021), doping with noble metal (Nawrot et al., 2020), combining with other semiconductors or organic polymers (Kiani et al., 2020). Among all the modification measures, combining with organic substances (especially conjugated polymers) shows great advantages owing to their excellent characteristics (Zhang et al., 2020). Polymers can provide a suitable cyberspace so as to limit the continuous growth of crystalline grain and prevent the particle aggregation. Moreover, the high stability, simple synthesis and good environmental compatibility of polymers are helpful for practical application (Chen et al., 2020; Zeng et al., 2021). Besides, the CdS/conjugated polymer nanocomposites possess better machinability and optical performance (Lee and Chang, 2019; Melinte et al., 2019).

Cellulose, as one of renewable resources on the Earth and the most abundant natural polymers (Tian Li et al., 2021), has been widely investigated as a modifier for semiconductors because of its distinctive properties, including biocompatibility, biodegradation, mechanical strength, and chemical stability (Jiang et al., 2019). Microcrystalline cellulose (MCC) is a kind of common organic conjugated polymers, which was obtained by purifying and depolymerizing cellulose. It possesses a lot of properties that eligible for modifying semiconductors, such as large surface area for interaction with water, water insolubility, high water absorption and retention capacity, good blinding nature, remarkable ability to prevent phase separation (Zhou et al., 2019; Amaly et al., 2022). Because of the above advantages, MCC has been considered as a promising material to modify semiconductor photocatalysts for enhanced water purification performance.

In this work, a simple and efficient ultrasound approach was designed to synthesize CdS/MCC nanocomposites. The photocatalytic activity of the products was tested based on the decoloration of MB aqueous solution under visible-light. The degradation mechanism of MB over CdS/MCC nanocomposite was also explored.

## Materials and Methods

### Materials

Microcrystalline cellulose (MCC), Cd(CH_3_CH_2_O)_2_·2H_2_O, thioacetamide (TAA), PVP (MW = 40,000 g/mol), methylene blue (MB), 1, 4–benzoquinone (BQ), triethanolamine (TEOA), and isopropanol (IPA), coumarin (COU) nitroblue tetrazolium (NBT), and ethanol were bought from Sinopharm Chemical Reagent Co., Ltd. The reactants and solvents were analytical grade and used with no further purification. Ultrapure water (resistivity 18.2 MΩ cm) was used in this study.

### Preparation of CdS/MCC Nanocomposites

The synthesis was conducted in SK3210HP 53 kHz Ultrasonic Cleaner (Shanghai KUDOS Ultrasonic Instrument Co., Ltd.). 0.2 g PVP and 0.1–0.5 g MCC were dissolved into 20 ml ethanol by sonication (18 W/cm^2^) for 30 min. Next 100 ml 0.28 mol/L Cd(CH_3_CH_2_O)_2_·2H_2_O aqueous solution was added and further ultrasonically treated for 30 min, subsequently 100 ml 0.2 mol/L TAA aqueous solution was introduced. Keep ultrasonic treatment for 3 h. The formed solid product was washed with ultrapure water and ethanol, then dried at 60°C for 12 h. Depending on the different MCC contents, the CdS/MCC composites were marked as CdS/MCC-3%, CdS/MCC-7%, CdS/MCC-10% and CdS/MCC-12%.

For comparison, CdS nanoparticles were synthesized according to the aforementioned procedures but in the absence of MCC.

### Characterization of the Prepared Photocatalyst

The compositions and microstructures of the CdS/MCC nanoparticles were analyzed by X-ray diffractometer (XRD, D8, Germany Bruker), field emission scanning electron microscopy (FE–SEM, S4800, Japan Hitachiltd, accelerating voltage 15 kV), and transmission electron microscopy (TEM, JEM–2100UHR, Japanese electronics, accelerating voltage 200 kV). Compositional analysis was carried out by energy-dispersive X–ray analysis (EDS). Nitrogen sorption measurements were performed with N_2_ at 77 K after degassing the samples at 300°C under vacuum for 3 h using a Quantachrome Quadra orb SI-MP porosimeter. The specific surface area was obtained by using the Brunauer–Emmett–Teller (BET) model to analyze the N_2_ adsorption equilibrium data. UV–Vis diffuse reflectance spectra (UV–Vis DRS) was performed by a UV–Vis spectrophotometer (UV–2600, Shimadzu Corporation).

### Photocatalytic Performance Tests

The photocatalytic performance of CdS/MCC nanocomposites synthesized were tested based on the degradation of MB in aqueous solution. 0.05 g CdS/MCC composites and 50 ml 10 mg/L MB solution were mixed by magnetic stirring for 30 min under dark condition to realize an adsorption/desorption equilibrium between the organic dye and photocatalyst. The photoreactor comprises a Xe arc lamp (1000 W) with a 400 nm optical filter, a cold trap to prevent the temperature rise of the reaction solution, and a set of 80 ml capacity cylindrical quartz reactors (Beijing Precise Technology Co., Ltd., PL-02). At the given intervals of irradiation time, 4 ml solution was fetched and filtered through a 0.45 μm filter to get rid of the photocatalyst, then the absorbance of the supernatant was measured at the wavelength of 664 nm using a Shanghai Lengguang Technology Co., Ltd. GS54T UV–Vis spectrophotometer. The degradation efficiency (η) of MB was calculated using the following :

$$\text{η}=\frac{{\text{A}}_{0}-\text{A}}{{\text{A}}_{0}}\times 100\text{\%}=\frac{{\text{C}}_{0}-\text{C}}{{\text{C}}_{0}}\times 100\text{\%}$$ (1)

Where *η* = the decolorization efficiency, C = the MB concentration at a certain irradiation time, and C_0_ = the MB concentration at the dark environment adsorption-desorption equilibrium.

## Results and Discussion

### Crystal Phase and Morphology Analysis

**FIGURE 1:**
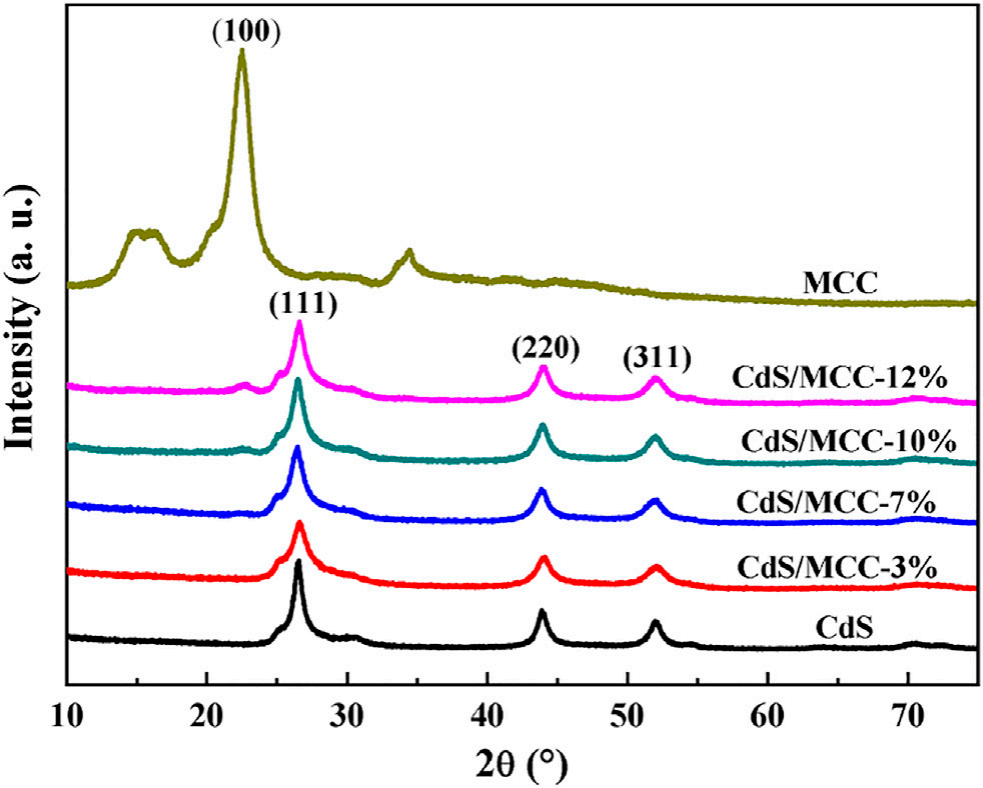
XRD patterns of CdS, MCC, and CdS/MCC composites with different contents of MCC.

Figure 1 displays the XRD patterns of the synthesized CdS/MCC composites with different contents of MCC, CdS and MCC. Compared with the standard XRD peaks of cubic phase CdS (JCPDS file No. 80–0019) and hexagonal phase CdS (JCPDS file No. 800–0006), the synthesized CdS comprised a mixture of major cubic phase and minor hexagonal phase. The peaks at 2θ values of 26.5°, 44.0°, and 52.1° were in turn assigned to the (111), (220), and (311) planes of cubic phase CdS. The weaker peaks at 2θ values of 26.7° and 28.3° corresponded to the (111) and (101) planes of hexagonal phase CdS (Kong et al., 2022), respectively. The XRD pattern of MCC showed sharp and high peaks, which revealed that the MCC itself has excellent crystallization. As for the various CdS/MCC composites, they display similar XRD spectra to mere CdS. Apparently, the XRD peaks of MCC did not appear in the XRD spectra of the CdS/MCC-3% and CdS/MCC-7% samples, which may be because that the minor amounts of MCC cannot be detected by the XRD equipment. When the MCC content is more than 10%, the significant (100) peak of MCC at a 2θ value of 22.5° begins to appear in the CdS/MCC composites. This confirmed that the MCC was successfully introduced to CdS and had formed CdS/MCC composites. Nevertheless, the introduction of MCC caused not shift of the XRD peak positions of CdS in the CdS/MCC composites, which suggested that MCC is not doped into the lattice of CdS nanocrystals.

**FIGURE 2:**
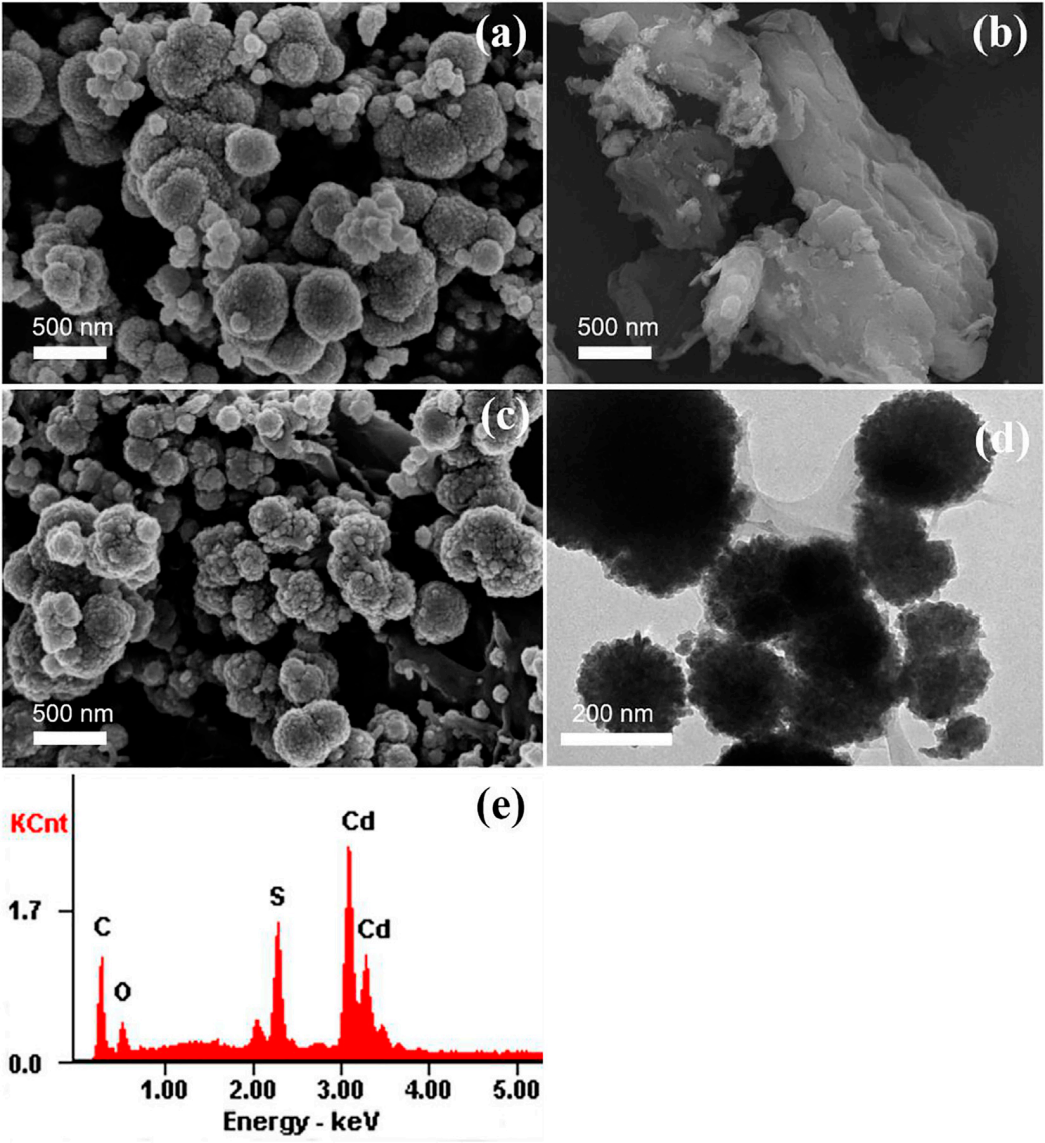
FE-SEM image of **(A)** CdS and **(B)** MCC; **(C)** FE-SEM image, **(D)** TEM image and **(E)** EDS pattern of CdS/MCC-10%.

The morphology, size and elemental compositions of the products were investigated using FE-SEM, TEM, and EDS. The SEM images of CdS, MCC and CdS/MCC-10% are shown in Figures 2A–C, respectively. The CdS and CdS/MCC-10% samples were both microsphere shape with the sizes around 100–500 nm. On the other hand, the MCC had a rod-like structure, and a large number of pleats were observed on its surface. When combined with MCC, the surface of CdS nanoparticles becomes more rough to a certain degree. The TEM image of CdS/MCC-10% can be seen in Figure 2D. MCC in the form of platelike spread around the CdS nanoparticles, forming a tight bonding interface. The corresponding EDS pattern (Figure 2E) of the as-prepared CdS/MCC-10% sample revealed that the nanocomposite contained the Cd, S, C, and O elements, and the atomic percentages of Cd, S, C, and O were 39.08, 37.82, 12.91, and 10.19%, respectively. The characteristic signals of C and O come from MCC, whereas S and Cd originate from CdS.

### Nitrogen Adsorption Analysis

**FIGURE 3:**
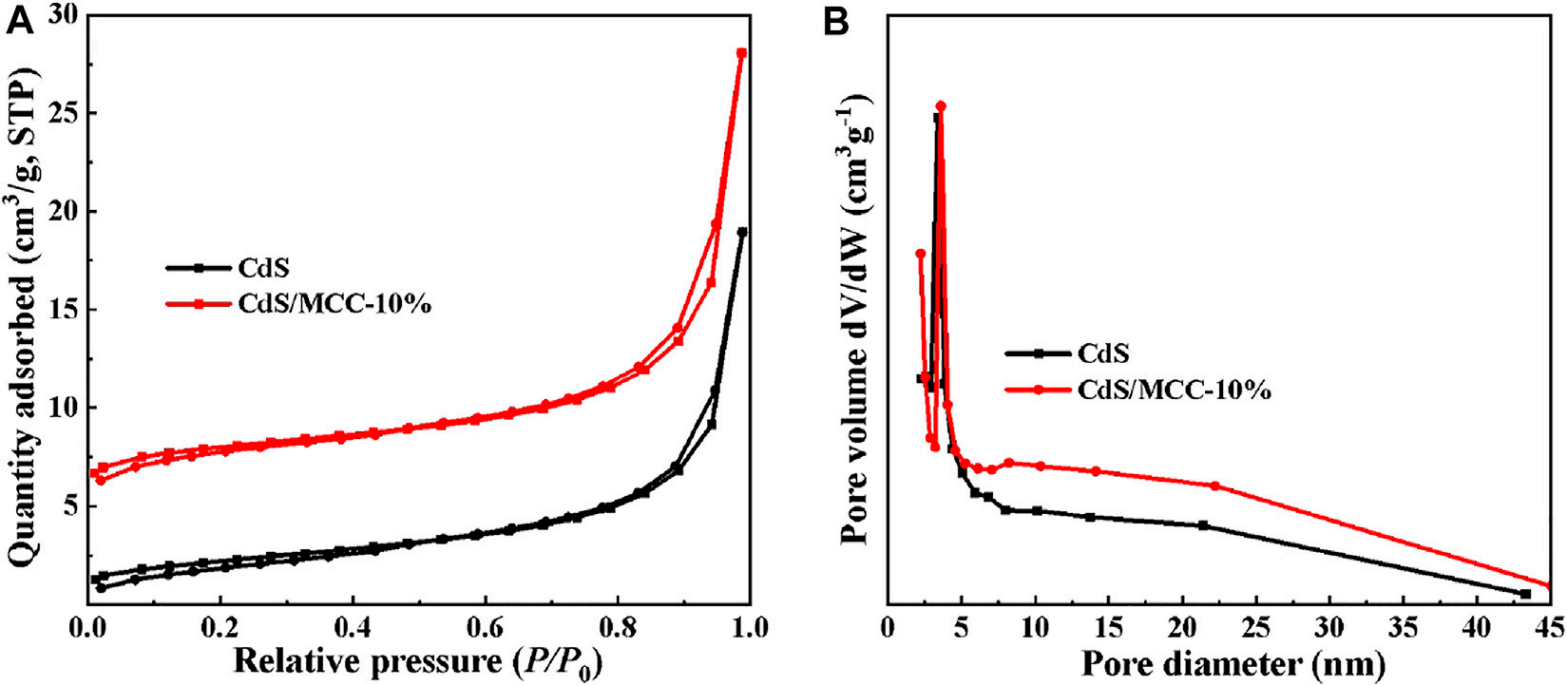
**(A)** N_2_ adsorption-desorption isotherms and **(B)** the corresponding pore-size distribution curves of CdS/MCC-10% and CdS.

Nitrogen adsorption tests at 77 K were performed to examine the porosity of the samples of pure CdS and CdS/MCC-10%. The BET specific surface areas of pure CdS and CdS/MCC-10% were 7.89 and 10.26 m^2^/g. Figures 3A,B show respectively the nitrogen adsorption-desorption isotherms and the corresponding pore-size distribution curves of the prepared products. The isotherms of the CdS/MCC-10% and CdS show similar hysteresis loops ranging at *P/P*_0_ = 0.8–1.0 and 0.9–1.0, respectively, which match the type IV on the basis of the IUPAC classification (You et al., 2021). The pore-size distribution curves of both products show some disorder with the pore-size distribution from 3 to 30 nm.

### UV-Vis DRS Spectra Analysis

**FIGURE 4:**
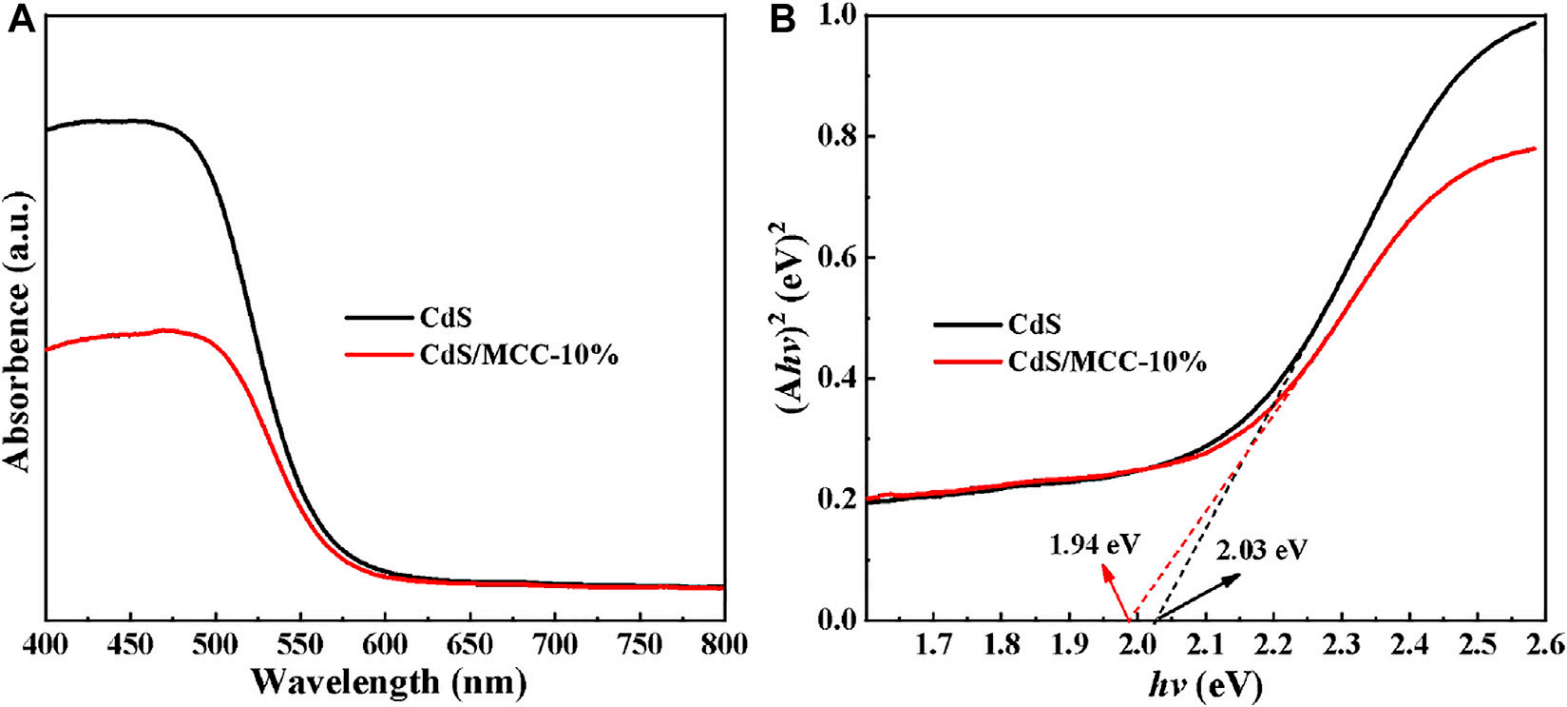
**(A)** UV–Vis DRS results and **(B)** Plots of (A*hv*)^2^ vs. *hv* to determine the E_g_ values of pure CdS and CdS/MCC-10%.

The UV–Vis DRS results of our prepared pure CdS and CdS/MCC samples were used to analyze their optical absorption properties and bandgap energies. Figure 4A illuminates that both samples exhibit obvious absorption of 400–550 nm visible-light. The bandgaps of the two samples are procured according to the Tauc’s plots in Figure 4B (Shkir et al., 2021). The determined E_g_ values of CdS and CdS/MCC are 1.94 and 2.03 eV, respectively. Compared with pure CdS, the narrower bandgap value of CdS/MCC-10% indicates that it can absorb a wider range of visible-light, which can be favorable for photocatalytic reactions (Zhao et al., 2021).

### Assessment of Photocatalytic Activity of CdS/MCC Composites

**FIGURE 5:**
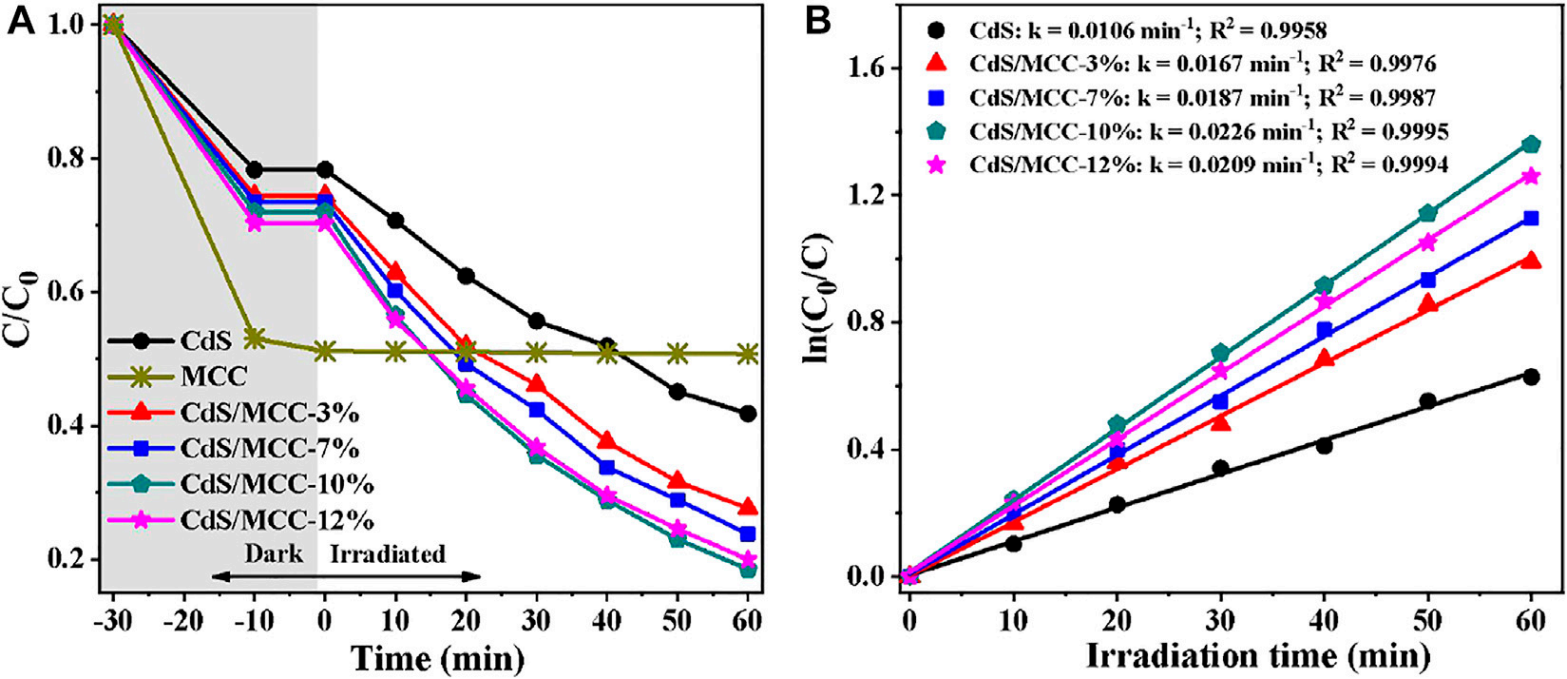
**(A)** Adsorption and photocatalytic decolorization of MB over prepared photocatalysts; **(B)** Corresponding kinetics plots for the photocatalytic MB decolorization reactions in **(A)**.

The photocatalytic activities of all the products were assessed for MB degradation under visible-light (λ > 400 nm) irradiation for 60 min. Figure 5A and Figure 5B displays the degradation of MB photocatalyzed by pure CdS, MCC and CdS/MCC composites under visible-light. From tte figures, MCC has strong adsorption capacity for MB, but almost no photocatalytic effect. The photocatalytic performance of CdS/MCC composites (especially the CdS/MCC-10% sample) improved significantly compared with CdS. When irradiated for 60 min, the CdS/MCC-10% sample has degraded 81.5% of the initial MB aqueous solution while the pure CdS degraded about 58.8%. When the MCC contents in the CdS/MCC composites were 3, 7, and 12%, the photodegradation rates of MB were lower than that over the CdS/MCC-10% sample. This suggests that MCC/CdS-10% had the highest visible-light photocatalytic activity compared with other CdS/MCC composites. Hence, there is an optimum MCC content for CdS/MCC composite to reach the most efficient visible-light photocatalysis. When the combined MCC was excess, the MCC sites may also function as photogenerated charge recombination centers. Accordingly, the excessive MCC sites can significantly reduce the amount of photogenerated charges and decrease the visible-light photocatalytic efficiency of CdS/MCC composites. Nevertheless, too less MCC content in the CdS/MCC composites resulted in less active sites, which also decreased the photocatalytic efficiency. Besides, the MB adsorption by CdS/MCC nanocomposites improved compared with pure CdS, which is consistent with the result of Nitrogen adsorption analysis.

### Photocatalytic Stability of Samples

**FIGURE 6:**
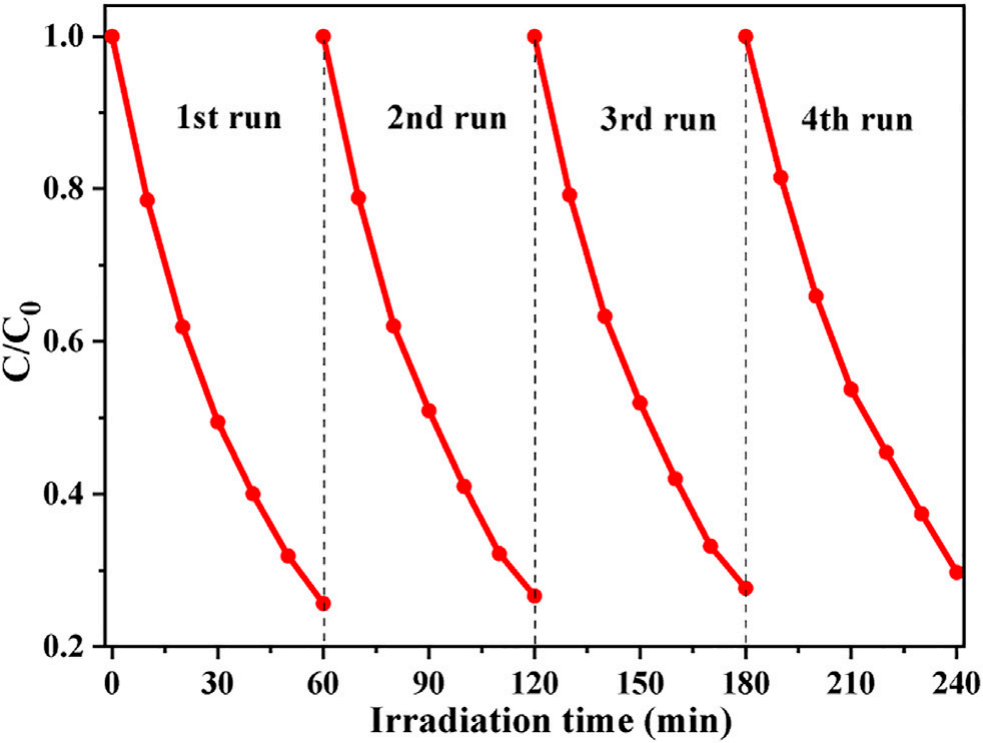
Repeated uses of CdS/MCC-10% in the photocatalytic degradation of MB.

For practical application, it is also important for the photocatalyst to have high photocatalytic stability (Ping Li et al., 2021). The photocatalytic stability of CdS/MCC-10% was further assessed in the MB decoloration reactions. The same photocatalytic process as above-mentioned was repeated for several times, but used 200 ml of MB and 200 mg of photocatalyst. After every cycle of reactions, the photocatalyst was collected, washed and used again, meanwhile the initial concentration of MB was maintained by injecting the stock solution of MB. As Figure 6 shows, the photocatalytic activity of CdS/MCC-10% had little decrease after the fourth run tests, which indicate that the CdS/MCC-10% photocatalyst is reusable for the environmental purification application.

### MB Degradation Mechanism Over CdS/MCC Composite Photocatalyst

**FIGURE 7:**
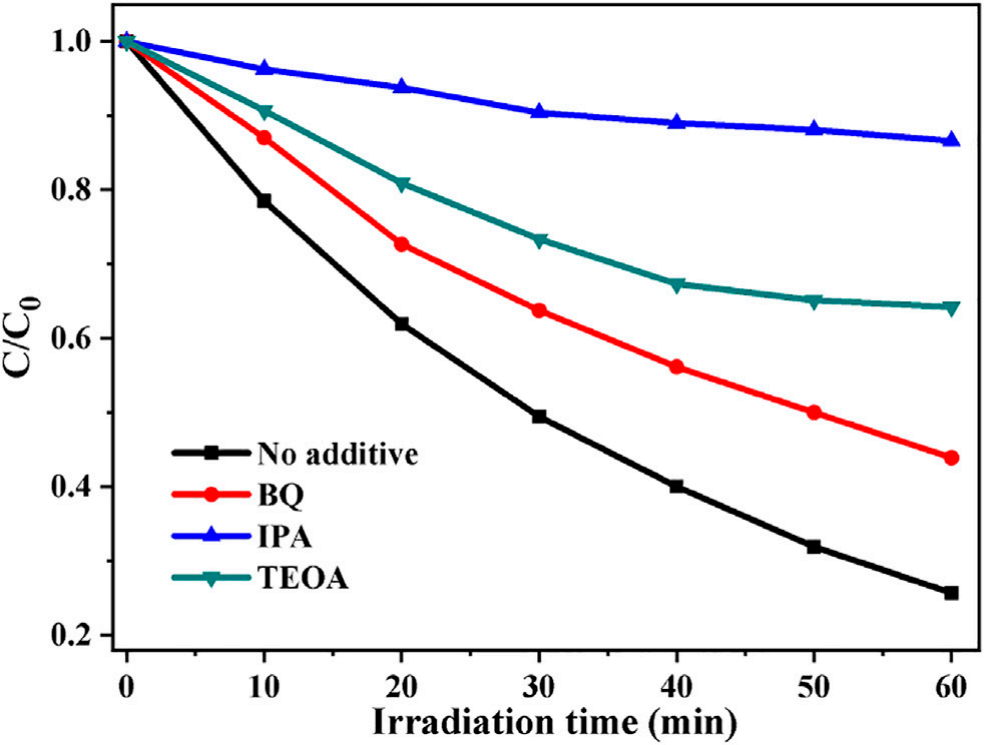
Photocatalytic MB degradation by CdS/MCC-10% with the addition of different scavengers.

To investigate the photocatalytic MB degradation mechanism by the CdS/MCC composite, the major reactive oxygen species involved in the degradation of MB were determined by free radical trapping experiments. The experimental procedure was the same as the photocatalytic activity test, but 50 ml of mixture of MB solution and scavenger, instead of 50 ml of MB solution was used. BQ (0.001 mol/L), TEOA (0.01 mol/L), and IPA (0.02 mol/L), were introduced into the reaction system as the scavengers for •O_2_^−^, h^+^, and •OH (Zhu et al., 2016; Gao et al., 2021), respectively. As can be clearly seen from Figure 7, the presence of TEOA, IPA and BQ all remarkably reduces the photocatalytic decolorization efficiency of MB under the same condition, which suggests that all •O_2_^−^, h^+^, and •OH participated in the decolorization reactions of MB. However, the photocatalytic degradation rate of MB after the addition of IPA is the slowest, indicating that •OH played a major role in the photocatalytic degradation of MB by CdS/MCC composite.

**FIGURE 8:**
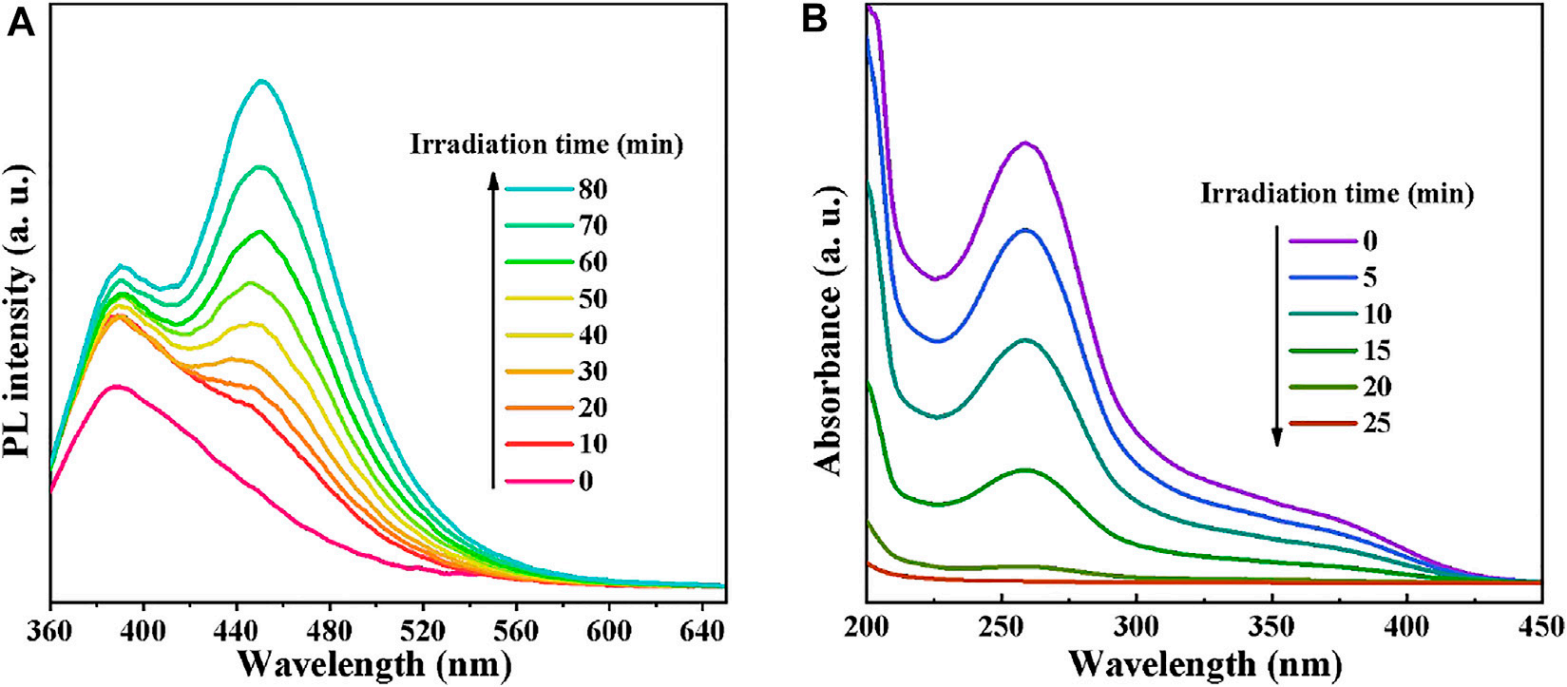
**(A)** PL intensities emitted by the mixture of CdS/MCC-10% and COU solution at different visible-light irradiation times; **(B)** The time-varying absorption spectra of NBT in the presence of CdS/MCC-10% and visible-light.

To verify the above experiment results, the generated •OH and •O_2_^−^ species in the photocatalytic process were inspected by COU photoluminescence probing and NBT transformation techniques, respectively. As •OH can react with COU to generate strongly fluorescent substance (7-hydroxycoumarin), the generation of •OH in the light-irradiated CdS/MCC-10% aqueous suspension was detected using fluorescent probe technique. In this experiment, a 50 ml COU solution (0.1 g/L) replaced the 50 ml MB solution. The supernatant was monitored directly using a fluorescence spectrophotometer (the excitation wavelength is 340 nm). The PL intensities emitted by the mixture of CdS/MCC-10% and COU solution at different visible-light irradiation times are shown in Figure 8A. Clearly, the PL intensity of the generated 7-hydroxycoumarin at about 453 nm became stronger as the irradiation time increased. These results indicated that the •OH radicals were produced and they might act as the predominant active oxygen species during the photocatalytic process (Wang et al., 2020). The •O_2_^−^ radicals in the mixture of 50 mg CdS/MCC-10% and 50 ml 0.01 mmol/L NBT solution under light irradiation were determined employing NBT (Shi et al., 2020; Du et al., 2021; Feng et al., 2021). The time-varying absorption spectra of NBT in the presence of CdS/MCC-10% and visible-light are shown in Figure 8B. The maximum absorbance of NBT at 260 nm decreased fastly as the irradiation time increased, indicating the formation of •O_2_^−^ in the CdS/MCC-10% system under visible-light.

**FIGURE 9:**
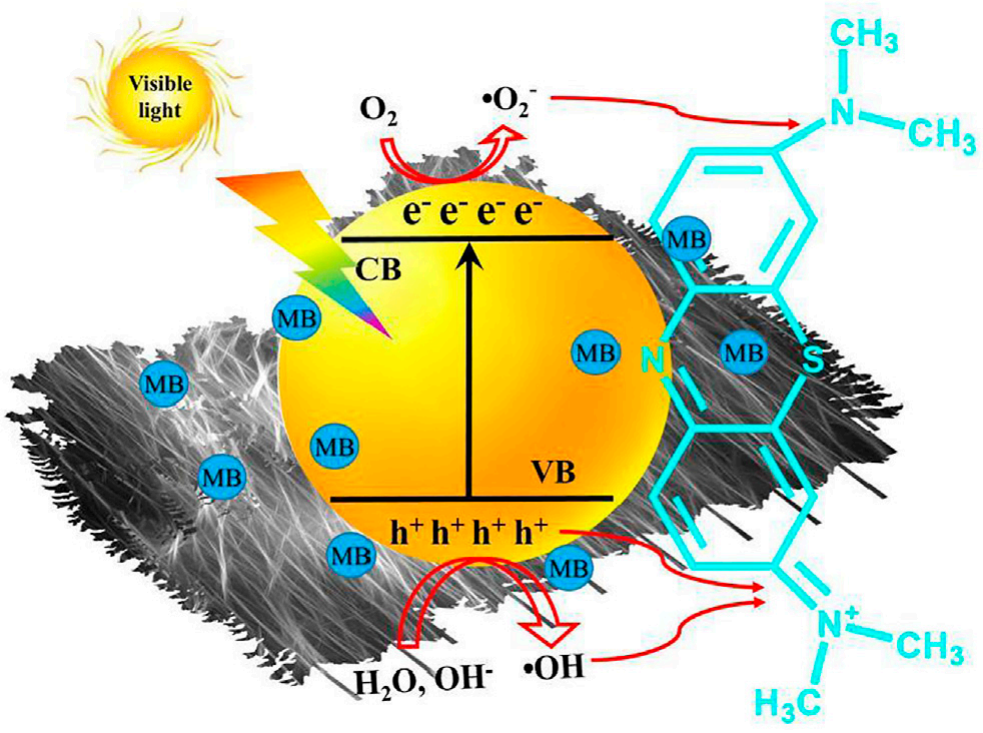
MB degradation mechanism over CdS/MCC composite photocatalyst.

Based on the above analysis, the photodegradation processes of MB over the CdS/MCC composite photocatalyst are interpreted in Figure 9. The photogenerated electrons and the photogenerated holes are produced respectively in the conduction band (CB) and valence band (VB) of CdS/MCC by excitation with visible-light (Eq. 2). Meanwhile a portion of the photogenerated charges would recombine. When MB molecules were adsorbed on the surface of CdS/MCC composite and excited, they would deliver electrons to the CB of CdS/MCC composites. The VB holes of CdS/MCC can oxidize MB (Eq. 3), or react with the surface bound OH^−^ (or by H_2_O) to generate •OH (Eqs 4, 5) (Liu et al., 2022). The •OH can attack the chromophoric structure and the diethylamino groups effectively, causing the cycloreversion of MB molecules (Eq. 6) (Sun et al., 2021) as well as final mineralization into CO_2_, H_2_O, and other inorganic substances. Besides, the highly reducing electrons in the CB of CdS/MCC can reduce O_2_ to •O_2_^−^ (Yao et al., 2020), which are also able to oxidize MB (Eqs 7, 8). The MCC can transfer electrons from its excited energy level to the CB of CdS, increasing the reactive oxygen species (•O_2_^−^) and reducing electron-hole recombination (Hasanpour et al., 2021). Moreover, the heterojunction formed between the two components of the synthesized CdS/MCC nanocomposite (Figure 2C) also favors the charge separation.

$$\text{CdS}+\text{hv}\to {\text{h}}^{+}+{\text{e}}^{-}$$ (2)

$$\text{MB}+{\text{h}}^{+}\to \text{oxide}\;\text{product}$$ (3)

$${\text{h}}^{+}+{\text{OH}}^{-}\to •\text{OH}$$ (4)

$${\text{h}}^{+}+{\text{H}}_{2}\text{O}\to •\text{OH}+{\text{H}}^{+}$$ (5)

•OH+MB→oxide product (6)

$${\text{e}}^{-}+{\text{O}}_{2}\to •{\text{O}}_{2}^{-}$$ (7)

$$•{\text{O}}_{2}^{-}+\text{MB}\to \text{oxide}\;\text{product}$$ (8)

## Conclusion

CdS/MCC nanocomposite photocatalyst has been successfully prepared by a mild and simple sonochemical method. In addition, the photocatalytic performance of the prepared products was also assessed. The results indicated that the synthesized CdS/MCC nanocomposite exhibited excellent photocatalytic activity, and the optimum content of MCC in the CdS/MCC nanocomposite was found to be 10%. When irradiated by visible-light for 60 min, CdS/MCC-10% can catalyze the degradation of 81.5% of MB, much higher than pure CdS. The boosted photocatalytic efficiency of CdS/MCC-10% was likely resulted from the tight junction between CdS and MCC and the larger surface area. Furthermore, the radicals capture experiments, NBT transformation and coumarin photoluminescence probing suggested that •OH was the dominant active oxygen species in the photocatalytic degradation of MB by CdS/MCC-10%. Our research provides evidence that the modification with MCC can efficiently improve the photocatalytic activity of semiconductors (such as CdS).

## Data Availability

The original contributions presented in the study are included in the article/Supplementary Material, further inquiries can be directed to the corresponding authors.
